# Construction and Verification of a Predictive Model for the Progression of Aortic Valve Calcification

**DOI:** 10.5334/gh.1473

**Published:** 2025-09-24

**Authors:** Zhen Guo, Zhenyu Xiong, Chaoguang Xu, Jingjing He, Shaozhao Zhang, Rihua Huang, Menghui Liu, Jiaying Li, Xinxue Liao, Xiaodong Zhuang

**Affiliations:** 1 Department of Cardiology, the First Affiliated Hospital, Sun Yat-Sen University, Guangzhou, China; 2 NHC Key Laboratory of Assisted Circulation and Vascular Diseases, Sun Yat-sen University, Guangzhou, China; 3 Institute of Guangdong Provincial Geriatrics, Guangdong Provincial People’s Hospital (Guangdong Academy of Medical Sciences), Southern Medical University, Guangzhou, China

**Keywords:** Aortic valve calcification, progression, predictive model, online platform

## Abstract

**Background:**

The primary objective of this study is to develop and validate a predictive model assessing the likelihood of disease progression in individuals with aortic valve calcification (AVC).

**Methods:**

For the second and third visits, 2,533 patients were followed up. They were randomly assigned to a train set and a validation set at a ratio of 7:3. After employing the Least Absolute Shrinkage and Selection Operator (LASSO) and multiple Cox regression to filter predictors, the selected variables were input into the Cox proportional risk model for model construction. Calibration curve, Consistency Index (C-index), Receiver Operating Characteristic (ROC) curve, and Decision Curve Analysis (DCA) were employed to validate the model. Patients were categorized into low- and high-risk groups based on the model’s predicted risk score, and survival analysis was conducted using Kaplan-Meier (K-M) plots. An online platform was used to enhance the clinical utility.

**Results:**

The incidence of AVC progression was 9.63%. LASSO-Cox regression analysis identified seven variables significantly correlated with AVC progression. In both the training and validation sets, the Area Under the Curve (AUC) and C-index of the prediction model exceeded 0.8. The calibration curve aligned closely with the diagonal line. Decision Curve Analysis (DCA) underscored the clinical application value of the model. Survival analysis demonstrated a significantly higher progression rate in the high-risk group compared to the low-risk group. The online platform visualized the probability of progression.

**Conclusion:**

The developed predictive model has proven reliability and accuracy in forecasting the 2-, 3-, and 4-year progression rates of patients with AVC. It offers a dependable framework for estimating progression and facilitating individualized comprehensive prevention strategies for individuals with AVC.

## Introduction

Aortic valve calcification (AVC) is a degenerative cardiovascular disease. It is more common in the elderly, and the prevalence increases sharply with age (1,2). The severity of AVC is closely related to the severity of aortic stenosis. When the disease progresses to severe aortic stenosis, the prognosis of patients is extremely poor and the mortality is high. The application rate of aortic valve replacement—the only treatment—is only about 60% (3). However, it should be noted that when the valve has significant calcification, patients with high AVC score have a higher incidence of perivalvular leakage after transcatheter aortic valve replacement (TAVR) (4,5).

The effect of AVC on cardiovascular events is significant. It can be used as an independent predictor in predicting the risk of cardiovascular and coronary events, independent of traditional risk factors and inflammatory biomarkers (6). Controlling AVC progression can slow the progression of aortic stenosis, but clinical trial studies are not optimistic, and studies of different targets have not resulted in significant remission of disease progression (7,8,9,10,11,12,13,14,15,16,17,18).

In summary, accurate prediction tools for AVC are urgently needed. The construction of a predictive model for the progression of AVC provides a prospect for active risk assessment. In this study, samples with two AVC scores in the MESA database were used to build a predictive model, search for factors affecting AVC progression, and evaluate the risk of progression of different influencing factors, which is conducive to early identification of high-risk patients and individualized treatment.

## Materials and Methods

### Queues and baseline checks

The Multi-Ethnic Study of Atherosclerosis (MESA) is a community-based, multicenter prospective cohort study sponsored by the National Heart, Lung, and Blood Institute. Baseline examinations were conducted between July 2000 and September 2002. Visit 2 took place from July 2002 to January 2004 and visit 3 took place from January 2004 to July 2005. Institutional review boards at each center approved the study, and all participants provided written informed consent (19). A complete description of the design of MESA has been published elsewhere (20,21). This manuscript was prepared using MESA Research Materials obtained from the National Heart, Lung, and Blood Institute (NHLBI) Biologic Specimen and Data Repository Information Coordinating Center and does not necessarily reflect the opinions or views of the MESA or the NHLBI.

Data for this study were obtained from the initial examination of the MESA cohort. Demographic details such as age, gender, race, education, income, smoking, drinking habits, and drug use were collected through questionnaires (22). Resting blood pressure was measured three times in a sitting position and the average of the second and third readings was recorded as systolic and diastolic blood pressure, along with heart rate. Hypertension was defined as systolic blood pressure ≥140 mmHg, diastolic blood pressure ≥90 mmHg, or the use of medications, combined with a self-reported diagnosis of hypertension. Diabetes classification included categories of no diabetes, impaired fasting glucose, and diabetes. Ankle-brachial index (ABI), calculated as the ratio of systolic pressure, was measured in the dorsal foot artery or posterior tibial artery to that in the brachial artery (23). Blood samples collected from participants fasting for 12 hours were used to measure and calculate fasting glucose (FG), total cholesterol (TC), triglycerides (TG), low-density lipoprotein cholesterol (LDL-C), high-density lipoprotein cholesterol (HDL-C), lipoprotein[a] (Lp[a]), interleukin-6 (IL-6), C-reactive protein (CRP), N-terminal pro-B-type natriuretic peptide (NT-proBNP), creatinine, and homocysteine (Hcy) (23). The estimated glomerular filtration rate (eGFR) for each sample was computed using the Chronic Kidney Disease Epidemiology Collaboration (CKD-EPI Creatinine Equation) (2021).

### Measurement of valve calcification

The Agatston score was employed to assess AVC through continuous CT scans in a semi-automatic manner. The reliability of this measurement was ensured through duplicate assessments, demonstrating a high average agreement both within and between observers (kappa >0.9). Aortic valve calcification measurements were conducted at baseline (n = 6814, 2000–2002) and subsequently at visit 2 (2002–2004) or visit 3 (2004–2006).

Aortic valve calcification was defined as any calcification observed in the aortic lobes (24). Progression of AVC was identified as a positive difference between two scans (25,26). At baseline, the distinction for coronary artery calcification, AVC, and mitral valve calcification focused solely on the presence or absence of calcification. A value of Agatston score = 0 indicated the absence of calcification, while Agatston score >0 signified the presence of calcification.

### Statistical method

We described baseline features by AVC progression. R software (version 4.3.0) was used for analysis in this study. The ‘corrgram’ package is to be used to analyze the correlation of all variables in the baseline. The MESA data set was randomly divided into two groups according to 7:3, constituting the train set and the validation set. The train set was used to construct the model, and the validation set population was used for the internal verification of the model. LASSO-Cox regression model combines LASSO regularization with Cox proportional risk model to reduce the dimensionality of high dimensional data sets. Using 10-fold cross-validation with an optimal penalty coefficient λ to achieve both variable identification and variable contraction, preventing overfitting, is valuable when dealing with multiple predictors that may be unrelated or redundant data sets. The ‘Glmnet’ package is to be used to screen progression-related variables. Risk scores for each sample were estimated by weighting Cox regression coefficients. At the same time, the C-index was calculated to determine the accuracy of the model constructed by the selected variables. Using the ‘survival’ package, all samples were classified as high and low risk based on the optimal threshold for the risk score, and progressive curves were described for different risk populations. The ‘ROC’ package was used to describe the ROC curve, assess the sensitivity and specificity of the model, and calculate the AUC value from the ROC curve. The ability of the model to predict disease progression was further verified in the validation set. The ‘rms’ package was used to develop a nomogram and calibrate the probability of the train and validation sets predicting disease progression for 2, 3, and 4 years. The ‘ggDCA’ package was used to plot DCA and evaluate the clinical utility of the model. The ‘Shiny’ package was used to build a visual website platform.

## Results

### Baseline characteristics

**Figure 1:**
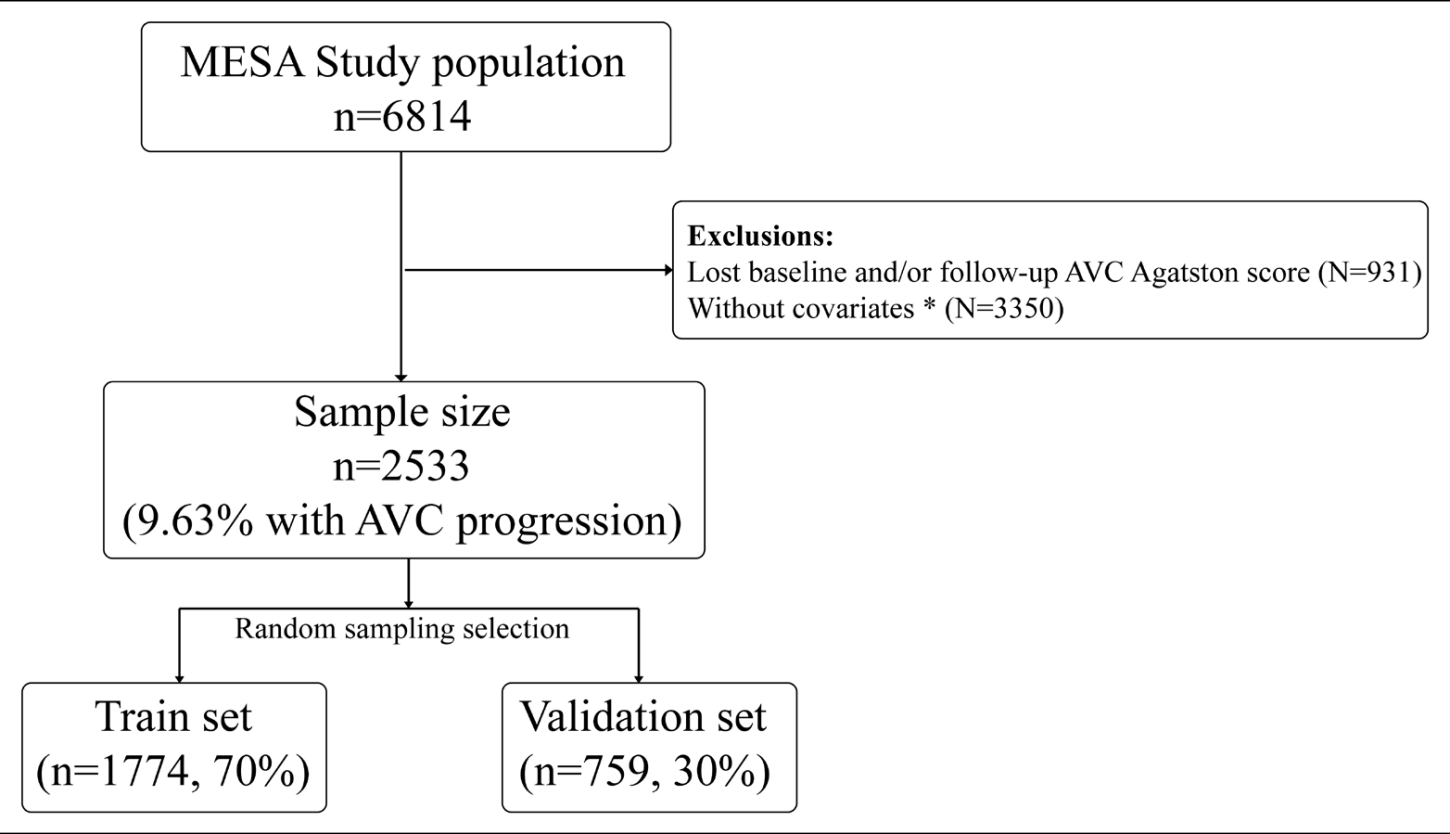
Flowchart of the included AVC patients. *Missing covariates: miss smoking (N = 14), miss drink (N = 22), miss income (N = 190), miss WHR (N = 1), miss ABI (N = 57), miss SBP (N = 331), miss RHR (N = 71), miss fast glucose (N = 114), miss Triglycerides (N = 1), miss LDL-C (N = 60), miss IL-6 (N = 224), miss CRP (N = 5), miss Lp[a] (N = 1571), miss NT-proBNP (N = 689).

Baseline data from the initial MESA examination, along with the number of participants undergoing CT scans during visits 2 and 3, are presented in Figure 1. The study comprised a total of 2,533 patients, of whom 244 exhibited progression at visits 2 and 3. Progression of AVC was determined by a difference of ≥0 between the AVC Agatston score at the second or third follow-up and the baseline AVC Agatston score.

**Figure 2:**
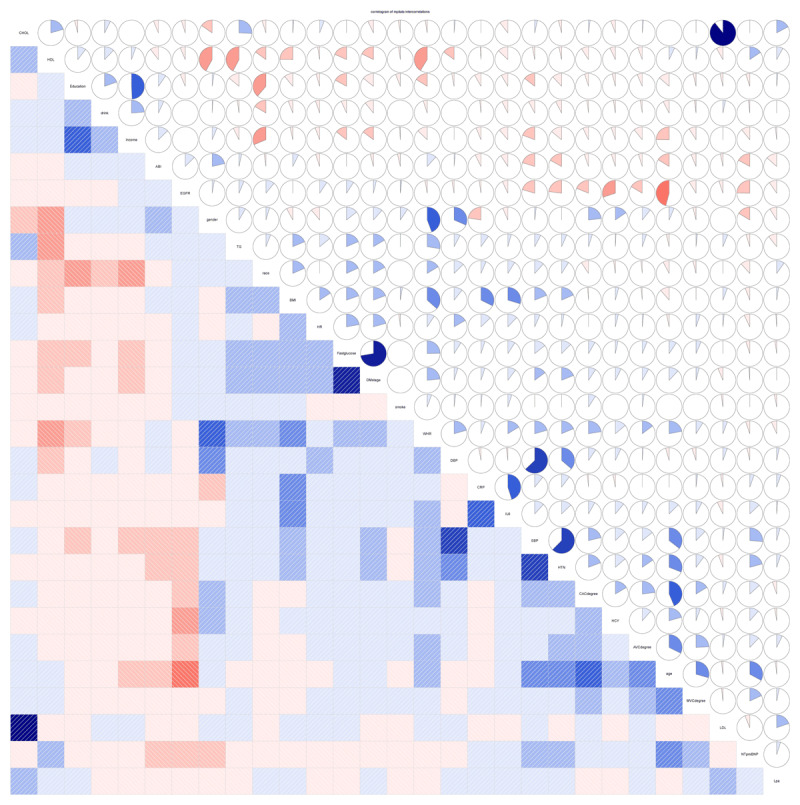
Correlation analysis of all variables at baseline.

The baseline characteristics of the population in this study include: age (61.372±10.284 years), gender (male 55.27%), ethnicity (Caucasian 45.08%, Chinese 7.78%, African American 25.54%, Hispanic 21.59%), median follow-up time (30.28 months), progression rate (9.63%) and so on. Stratified by progression status, Table 1 illustrates the baseline clinical features of all patients. The mean AVC at baseline was 119.152 ± 290.947 Agatston units (AU) in those who progressed, and 13.195 ± 115.153 AU in those who did not. Among the 2,227 patients without AVC at baseline, only 86 exhibited progression, with a mean longitudinal change of 18.032 ± 28.895 AU/year. Of the 306 patients with positive AVC at baseline, 158 developed AVC, experiencing a mean longitudinal change of 58.980 ± 130.309 AU/year, while the remaining 148 patients showed no progression, with a mean longitudinal change of –41.473 ± 120.752 AU/year. Pairwise correlations for these baseline measures are depicted in Figure 2.

**Table 1:** Baseline characteristics of progression and non-progression groups.


	LEVEL	OVERALL	NON-PROGRESSION	PROGRESSION	*p*

	n	2533	2289	244	

Age		61.37 ± 10.28	60.61 ± 10.15	68.55 ± 8.71	<0.001

Gender (%)	Female	1133 (44.7)	1055 (46.1)	78 (32.0)	<0.001

	Male	1400 (55.3)	1234 (53.9)	166 (68.0)	

Race (%)	Caucasian	1142 (45.1)	1019 (44.5)	123 (50.4)	0.055

	Chinese	197 (7.8)	183 (8.0)	14 (5.7)	

	African American	647 (25.5)	599 (26.2)	48 (19.7)	

	Hispanic	547 (21.6)	488 (21.3)	59 (24.2)	

Smoke (%)	No	1114 (44.0)	1018 (44.5)	96 (39.3)	0.143

	Yes	1419 (56.0)	1271 (55.5)	148 (60.7)	

Drink (%)	No	744 (29.4)	659 (28.8)	85 (34.8)	0.058

	Yes	1789 (70.6)	1630 (71.2)	159 (65.2)	

Education (%)	Less than high school education	357 (14.1)	314 (13.7)	43 (17.6)	0.018

	College education	1021 (40.3)	911 (39.8)	110 (45.1)	

	Graduate school Education	1155 (45.6)	1064 (46.5)	91 (37.3)	

Income	Income < 25,000/year	705 (27.8)	610 (26.7)	95 (38.9)	<0.001

	Income > 50,000 and ≤ 100,000/year	1408 (55.6)	1287 (56.2)	121 (49.6)	

	Income > 100,000/year	420 (16.6)	392 (17.1)	28 (11.5)	

BMI (kg/m^2^)		28.21 ± 5.31	28.20 ± 5.36	28.25 ± 4.84	0.885

WHR		0.92 ± 0.08	0.92 ± 0.08	0.96 ± 0.07	<0.001

ABI		1.13 ± 0.11	1.13 ± 0.11	1.11 ±0.13	0.008

Hypertension	No	1555 (61.4)	1449 (63.3)	106 (43.4)	<0.001

	Yes	978 (38.6)	840 (36.7)	138 (56.6)	

Diabetes stage	Normal	1946 (76.8)	1782 (77.9)	164 (67.2)	<0.001

	Impaired fasting glucose	356 (14.1)	313 (13.7)	43 (17.6)	

	Diabetes	231 (9.1)	194 (8.5)	37 (15.2)	

SBP (mmHg)		125.39 ± 20.36	124.60 ± 20.08	132.71 ± 21.49	<0.001

DBP (mmHg)		72.45 ± 10.21	72.39 ± 10.21	72.97 ± 10.12	0.399

Fastglucose (mg/dL)		95.02 ± 25.53	94.41 ± 24.59	100.86 ± 32.55	<0.001

Triglycerides (mg/dL)		123.95 ± 66.52	123.10 ± 66.78	131.92 ± 63.60	0.045

LDL-C (mg/dL)		119.34 ± 30.86	119.15 ± 30.76	121.16 ± 31.81	0.333

HDL-C (mg/dL)		51.21 ± 15.17	51.45 ± 15.04	48.91 ± 16.26	0.013

Total cholesterol (mg/dL)		195.34 ± 34.09	195.23 ± 33.94	196.43 ± 35.52	0.601

IL-6 (pg/mL)		1.53 ± 1.18	1.51 ± 1.16	1.78 ± 1.34	<0.001

CRP (mg/L)		3.48 ± 4.76	3.45 ± 4.70	3.70 ± 5.36	0.453

Lipoprotein[a] (mg/dL)		28.51 ± 30.83	27.78 ± 30.54	35.34 ± 32.72	<0.001

NT-proBNP (pg/mL)		89.66 ± 129.88	85.90 ± 117.38	124.94 ± 211.36	<0.001

EGFR (mL/min/1.73 m^2^)		81.67 ± 15.85	82.30 ±15.56	75.80 ± 17.30	<0.001

Resting heart rate (beats/min)		62.43 ± 9.43	62.31 ± 9.36	63.57 ± 10.00	0.047

HCY (umol/L)		9.37 ± 3.89	9.26 ± 3.83	10.41 ± 4.29	<0.001

CAC degree	No	1335 (52.7)	1271 (55.5)	64 (26.2)	<0.001

	Yes	1198 (47.3)	1018 (44.5)	180 (73.8)	

AVC degree	No	2227 (87.9)	2141 (93.5)	86 (35.3)	<0.001

	Yes	306 (12.1)	148 (6.5)	158 (64.8)	

MVC degree	No	2316 (91.4)	2121 (92.7)	195 (79.9)	<0.001

	Yes	217 (8.6)	168 (7.3)	49 (20.1)	

CAC score at exam 2 or 3		137.01 ± 377.64	112.74 ± 332.63	364.68 ± 621.82	<0.001

AVC score at exam 2 or 3		23.40 ± 145.20	13.20 ± 115.15	119.15 ± 290.95	<0.001

MVC score at exam 2 or 3		37.58 ± 374.37	34.97 ± 381.13	62.07 ± 303.29	0.283


### Progression data

The median follow-up duration for the study was 30.28 months. By the conclusion of the follow-up period, 244 individuals exhibited progression, while 2,289 individuals did not. Cumulative progression rates at years 1, 2, 3, 4, and 5 years were observed as 0% (0/1,121), 8.30% (93/1,121), 9.03% (153/1,694), 9.64% (241/2,500), and 9.63% (244/2,533), respectively.

### Prediction model based on LASSO-Cox regression

**Figure 3:**
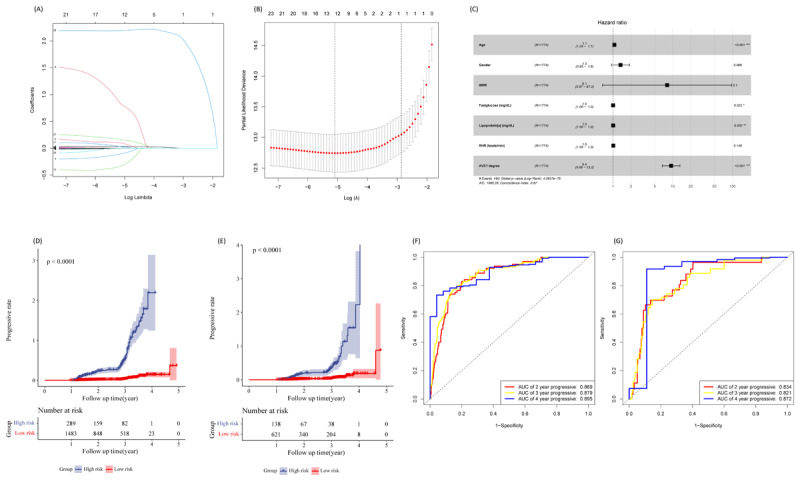
Construction of LASSO-Cox regression model. **(A, B, C)** LASSO Cox analysis identified seven variables most correlated to overall progression in verification set and train set. **(D, E, F, G)** Kaplan–Meier curves of overall survival based on the model in verification set and train set and ROC curve analysis of the model.

Patients were randomly assigned to a train set and a validation set at a ratio of 7:3. The comparison of most included variables between the two groups revealed no statistically significant differences (p > 0.05) (Table S1). LASSO regression was applied for variable screening, and the variation characteristics of these variable coefficients are depicted in Figures 3A and 3B. A 10-fold cross-validation was implemented to analyze variables, indicating an optimal lambda value logarithmically around -5, where 12 variables (age, gender, drinking, waist-to-hip ratio (WHR), ankle-brachial index, hypertension, fasting glucose, HDL-C, lipoprotein[a], heart rate, CAC degree, AVC degree) yielded the lowest prediction error.

To address excessive variables and prevent overfitting, Akaike Information Criterion (AIC) was employed, resulting in the selection of seven variables with excellent predictive performance. These variables included age, sex, waist-to-hip ratio, fasting glucose, Lp[a], resting heart rate, and the presence or absence of AVC at baseline. A Cox regression model was constructed based on the parameters derived from LASSO regression screening (Figure 3C and Table 2). The C-index of the model was determined to be 0.869 (standard error = 0.014).

**Table 2:** The weight of the selected predictor.


	HR	95CI	P_VALUE

Age	1.056	1.038–1.075	<0.001

Gender	1.338	0.947–1.890	0.099

WHR	8.059	0.668–97.180	0.100

Fastglucose (mg/dL)	1.004	1.001–1.008	0.022

Lipoprotein[a] (mg/dL)	1.007	1.003–1.012	0.002

RHR (beats/min)	1.011	0.996–1.026	0.146

AVC degree	9.417	6.694–13.247	<0.001


The LASSO-Cox regression analysis was utilized to compute the risk score for each sample in the training set. The survminer package facilitated the determination of the optimal threshold to classify patients in the training set into high-risk and low-risk groups (Figure 3D). Kaplan-Meier analysis demonstrated a higher progression rate in the high-risk group. ROC curves were employed to assess the predictive performance of variables in the training set, revealing high accuracy and sensitivity in predicting 2-year, 3-year, and 4-year progression rates (AUC2 = 0.869; AUC3 = 0.879; AUC4 = 0.895) (Figure 3E).

The following formula illustrates how to calculate the 2-year, 3-year, and 4-year risk of AVC progression:

2 year progressive risk=0.9880362^exp(riskscore)

3 year progressive risk=0.9725802^exp(riskscore)

4 year progressive risk=0.8618241^exp(riskscore)

Riskscore=0.054447*age+0.29085*gender+2.86739*WHR+0.004458*Fastglucose+0.007181*Lpa+0.010994*RHR+2.242489*AVC degree

The validation set was utilized to substantiate the predictive efficacy of the model, applying the same formula for risk calculation. Using the identical approach, patients were categorized into high-risk and low-risk groups (Figure 3F). Notably, in the validation set, the main results are consistent with the training set (Figure 3G).

### Calibration and clinical application of LASSO-Cox regression model

**Figure 4:**
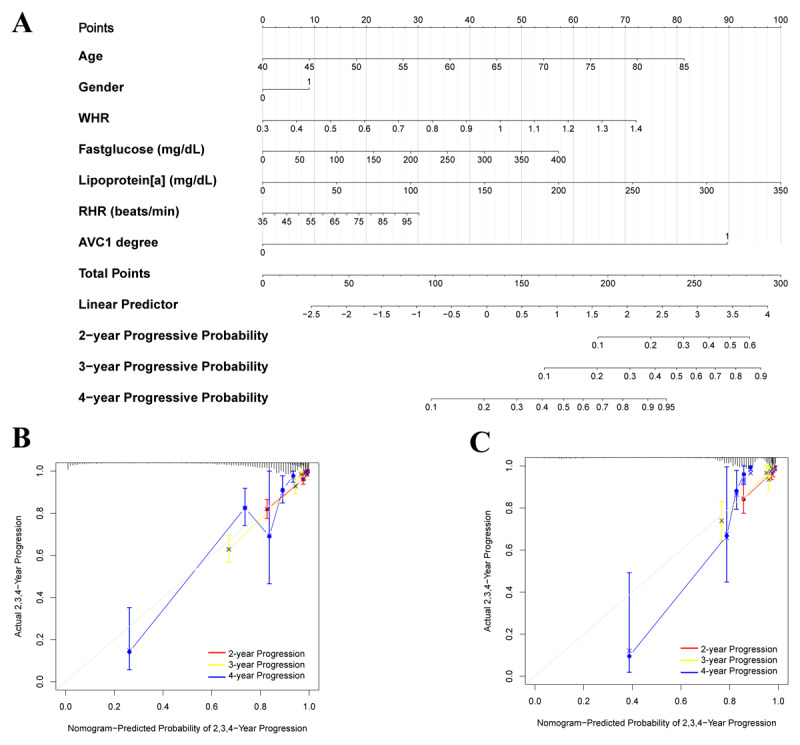
Construction of the nomogram model. **(A)** Nomogram model for predicting the probability of 2-, 3- and 4-year progressive rate. **(B)** Calibration plots of the nomogram for predicting the probability of 2-, 3- and 4-year progressive rate in train set. **(C)** Calibration plots of the nomogram for predicting the probability of 2-, 3- and 4-year progressive rate in verification set.

**Figure 5:**
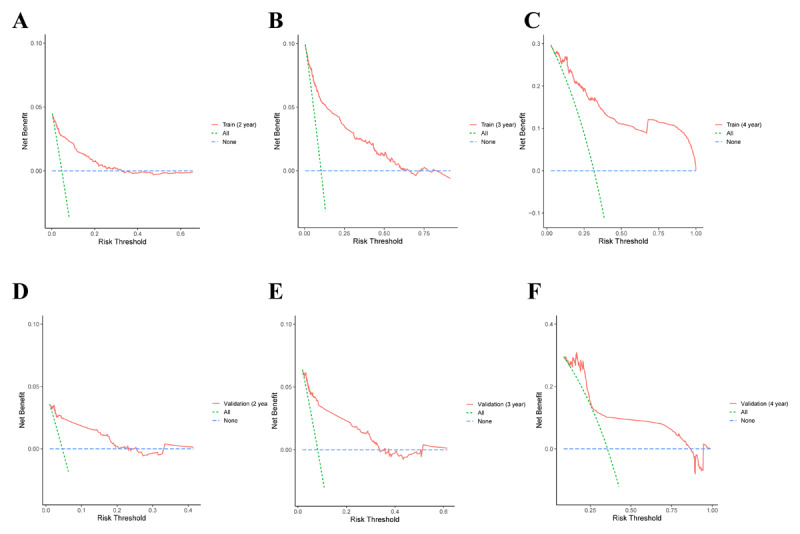
Decision curve analysis of prediction probability of training set and verification set at 2, 3, and 4 years.

A nomogram for predicting the progression of AVC was developed based on the seven selected variables (Figure 4A). Calibration curves illustrating the performance of the models in predicting progression at 2, 3, and 4 years demonstrated commendable concordance between predictions and observations across both the training and validation sets (Figures 4B and 4C). Additionally, the DCA curve further exhibited a strong agreement between the predicted probability of disease progression from the nomogram and the actual probability (Figure 5).

To enhance the clinical utility of the nomogram, we have provided an online platform (https://avc1.shinyapps.io/DynNomapp/). This tool allows for the quick estimation of the probability of progression in patients with mild to moderate AVC by adjusting variable parameters (example was shown in Figure S1).

## Discussion

The worldwide prevalence of AVC increases significantly with age, and individual progression varies greatly. When the disease has progressed to the point of aortic stenosis, especially severe stenosis, the only treatment option is aortic valve replacement. Therefore, there is a great need to identify and control disease progression in its early stages. At present, there is no accurate prediction tool for predicting the progression of AVC. In this study, we constructed a prediction model with good consistency and high accuracy using seven variables. In demonstrating clinical efficacy, our nomogram and online platform were able to accurately predict the progression of AVC.

In previous studies, similar to traditional cardiovascular events, older age and males were clear risk factors for aortic valve development and progression.

The oxidized phospholipids (OxPL) content of lipoprotein[a] plays an important role in valve calcification. In a study by Kang H Zheng et al., elevated lipoprotein[a] was associated with 2–3 times faster progression of valve calcification in people with an average age of 70 years (27,28). However, in another long-term study (median follow-up of 14 years), Lp[a] was associated with new AVC but not with AVC progression, that is, driving the initiation of aortic valve disease but not advancing progression (29).

Resting heart rate (RHR) is an independent risk factor for cardiovascular disease (CVD) in patients with and without heart disease. High RHR leads to increased blood pressure, vascular stiffness, endothelial dysfunction, and inflammation activation by increasing sympathetic nerve activity. Mechanical strain can initiate and accelerate the aggregation of aortic valve stromal cells, leading to calcification deposits (30). In a large sample of 5,498 patients, high RHR was associated with valvular calcification, especially AVC progression, but not with traditional CVD risk factors (30).

Obesity is associated with metabolic abnormalities. But studies on the association between body mass index (BMI) and calcifying aortic valve disease have shown conflicting results. Several small cross-sectional studies focusing on AVC have shown that weight loss does not delay the progression of aortic stenosis, possibly because the distinction between disease onset and progression is not clear (31). But in our study, an interesting finding was that BMI was not a risk factor for predicting the progression of valve calcification, but waist-to-hip ratio was. Waist-to-hip ratio emphasizes abdominal fat, demonstrating the necessity of distinguishing individual differences in metabolic status, which is consistent with previous studies on the association between aortic stenosis and metabolic syndrome (32,33).

Diabetes or hyperglycemia are key risk factors for CVD and play an important role in initiating the development and progression of valve disease. Hyperglycemia promotes aortic valve fibrosis and calcification (34,35). Currently, there are limited studies on hyperglycemia and valve calcification, but in some prospective studies on aortic stenosis, diabetes mellitus is associated with an increased risk of aortic stenosis (36,37).

The baseline AVC Agatston score is a marker of a high rate of progression. Patients with high Agatston scores at baseline themselves had a higher number of clinical factors that contributed to high calcification levels at baseline, and these factors continued to play a role during progression (38).

Admittedly, there are several limitations to our study. First, baseline characteristics showed significant differences in some variables between the progressive and non-progressive populations. This is unavoidable because of the low proportion of people who progress. However, these differences had no significant effect on the results. Second, our analysis assumes that the risk is homogeneous in the progressive and non-progressive populations, where progress from 0 to Agatston score >0 in the progressive population may be influenced by different factors than in the population with Agatston score >0 at baseline and further progress. We analyzed the baselines of the two, where some variables showed significant differences (Table S2). In previous studies, both were defined as progression, and we stuck with that definition. While the population with Agatston score >0 at baseline and further progress was defined as progress, the sample size was too small, and the accuracy of the model could not be guaranteed.

## Conclusion

In our study, we used aortic valve calcification samples in the MESA database to screen out seven key variables, including age, gender, waist-to-hip ratio, fasting glucose, lipoprotein[a], resting heart rate, and presence or absence of AVC at baseline, affecting the progression of calcification through LASSO-Cox regression analysis, and built a prediction model. At the same time, we established an online platform to accurately display the risk of progression, identify the high-risk population in the early stage of the disease, and comprehensively manage the risk factors, so as to delay the progression of AVC.

## Data Accessibility Statement

The data utilized in this study were obtained from publicly accessible resources, specifically from the MESA database, which can be accessed at https://www.mesa-nhlbi.org/. We confirm that our use of the MESA aortic-valve-calcification (AVC) data supplied by BioLINCC strictly adheres to the original Data Use Agreement signed between our team and BioLINCC. That agreement explicitly permits us to use the data for the purposes of this study, including the publication of related scientific papers.

## Additional File

The additional file for this article can be found as follows:

10.5334/gh.1473.s1 Supplementary file. Figure S1 and Tables S1 to S2.
