# Supplementary material for: Construction and Verification of a Predictive Model for the Progression of Aortic Valve Calcification
Source: Glob Heart. 2025 Sep 24;20(1):84. doi: 10.5334/gh.1473 (PMC12466327; doi:10.5334/gh.1473)
Supplement: Supplementary file. — Figure S1 and Tables S1 to S2. [file gh-20-1-1473-s1.zip › gh-1473_zhuang-s1/Table 1.docx]

Table 1. Baseline characteristics of progression and non-progression groups.

|  | level | Overall | Non-progression | Progression | *p* |
| --- | --- | --- | --- | --- | --- |
|  | n | 2533 | 2289 | 244 |  |
| Age |  | 61.37 ± 10.28 | 60.61 ± 10.15 | 68.55 ± 8.71 | <0.001 |
| Gender (%) | Female | 1133 (44.7) | 1055 (46.1) | 78 (32.0) | <0.001 |
|  | Male | 1400 (55.3) | 1234 (53.9) | 166 (68.0) |  |
| Race (%) | Caucasian | 1142 (45.1) | 1019 (44.5) | 123 (50.4) | 0.055 |
|  | Chinese | 197 (7.8) | 183 (8.0) | 14 (5.7) |  |
|  | African American | 647 (25.5) | 599 (26.2) | 48 (19.7) |  |
|  | Hispanic | 547 (21.6) | 488 (21.3) | 59 (24.2) |  |
| Smoke (%) | No | 1114 (44.0) | 1018 (44.5) | 96 (39.3) | 0.143 |
|  | Yes | 1419 (56.0) | 1271 (55.5) | 148 (60.7) |  |
| Drink (%) | No | 744 (29.4) | 659 (28.8) | 85 (34.8) | 0.058 |
|  | Yes | 1789 (70.6) | 1630 (71.2) | 159 (65.2) |  |
| Education (%) | Less than high School Education | 357 (14.1) | 314 (13.7) | 43 (17.6) | 0.018 |
|  | College Education | 1021 (40.3) | 911 (39.8) | 110 (45.1) |  |
|  | Graduate School Education | 1155 (45.6) | 1064 (46.5) | 91 (37.3) |  |
| Income | Income < 25,000/year | 705 (27.8) | 610 (26.7) | 95 (38.9) | <0.001 |
|  | Income > 50,000 and ≤ 100,000/year | 1408 (55.6) | 1287 (56.2) | 121 (49.6) |  |
|  | Income > 100,000/year | 420 (16.6) | 392 (17.1) | 28 (11.5) |  |
| BMI (kg/m2) |  | 28.21 ± 5.31 | 28.20 ± 5.36 | 28.25 ± 4.84 | 0.885 |
| WHR |  | 0.92 ± 0.08 | 0.92 ± 0.08 | 0.96 ± 0.07 | <0.001 |
| ABI |  | 1.13 ± 0.11 | 1.13 ± 0.11 | 1.11 ±0.13 | 0.008 |
| Hypertension | No | 1555 (61.4) | 1449 (63.3) | 106 (43.4) | <0.001 |
|  | Yes | 978 (38.6) | 840 (36.7) | 138 (56.6) |  |
| Diabetes_stage | Normal | 1946 (76.8) | 1782 (77.9) | 164 (67.2) | <0.001 |
|  | Impaired fasting glucose | 356 (14.1) | 313 (13.7) | 43 (17.6) |  |
|  | Diabetes | 231 (9.1) | 194 (8.5) | 37 (15.2) |  |
| SBP (mmHg) |  | 125.39 ± 20.36 | 124.60 ± 20.08 | 132.71 ± 21.49 | <0.001 |
| DBP (mmHg) |  | 72.45 ± 10.21 | 72.39 ± 10.21 | 72.97 ± 10.12 | 0.399 |
| Fastglucose (mg/dL) |  | 95.02 ± 25.53 | 94.41 ± 24.59 | 100.86 ± 32.55 | <0.001 |
| Triglycerides (mg/dL) |  | 123.95 ± 66.52 | 123.10 ± 66.78 | 131.92 ± 63.60 | 0.045 |
| LDL-C (mg/dL) |  | 119.34 ± 30.86 | 119.15 ± 30.76 | 121.16 ± 31.81 | 0.333 |
| HDL-C (mg/dL) |  | 51.21 ± 15.17 | 51.45 ± 15.04 | 48.91 ± 16.26 | 0.013 |
| Total cholesterol (mg/dL) |  | 195.34 ± 34.09 | 195.23 ± 33.94 | 196.43 ± 35.52 | 0.601 |
| IL-6 (pg/mL) |  | 1.53 ± 1.18 | 1.51 ± 1.16 | 1.78 ± 1.34 | <0.001 |
| CRP (mg/L) |  | 3.48 ± 4.76 | 3.45 ± 4.70 | 3.70 ± 5.36 | 0.453 |
| Lipoprotein[a] (mg/dL) |  | 28.51 ± 30.83 | 27.78 ± 30.54 | 35.34 ± 32.72 | <0.001 |
| NT-proBNP (pg/mL) |  | 89.66 ± 129.88 | 85.90 ± 117.38 | 124.94 ± 211.36 | <0.001 |
| EGFR (mL/min/1.73 m2) |  | 81.67 ± 15.85 | 82.30 ±15.56 | 75.80 ± 17.30 | <0.001 |
| Resting heart rate (beats/min) |  | 62.43 ± 9.43 | 62.31 ± 9.36 | 63.57 ± 10.00 | 0.047 |
| HCY (umol/L) |  | 9.37 ± 3.89 | 9.26 ± 3.83 | 10.41 ± 4.29 | <0.001 |
| CAC degree | No | 1335 (52.7) | 1271 (55.5) | 64 (26.2) | <0.001 |
|  | Yes | 1198 (47.3) | 1018 (44.5) | 180 (73.8) |  |
| AVC degree | No | 2227 (87.9) | 2141 (93.5) | 86 (35.3) | <0.001 |
|  | Yes | 306 (12.1) | 148 (6.5) | 158 (64.8) |  |
| MVC degree | No | 2316 (91.4) | 2121 (92.7) | 195 (79.9) | <0.001 |
|  | Yes | 217 (8.6) | 168 (7.3) | 49 (20.1) |  |
| CAC score at exam 2 or 3 |  | 137.01 ± 377.64 | 112.74 ± 332.63 | 364.68 ± 621.82 | <0.001 |
| AVC score at exam 2 or 3 |  | 23.40 ± 145.20 | 13.20 ± 115.15 | 119.15 ± 290.95 | <0.001 |
| MVC score at exam 2 or 3 |  | 37.58 ± 374.37 | 34.97 ± 381.13 | 62.07 ± 303.29 | 0.283 |

Data are shown as mean ± SD or n (%).
