# Supplementary material for: Construction and Verification of a Predictive Model for the Progression of Aortic Valve Calcification
Source: Glob Heart. 2025 Sep 24;20(1):84. doi: 10.5334/gh.1473 (PMC12466327; doi:10.5334/gh.1473)
Supplement: Supplementary file. — Figure S1 and Tables S1 to S2. [file gh-20-1-1473-s1.zip › gh-1473_zhuang-s1/Table 2.docx]

Table 2. The weight of the selected predictor.

|  | HR | 95CI | P_value |
| --- | --- | --- | --- |
| Age | 1.056 | 1.038-1.075 | <0.001 |
| Gender | 1.338 | 0.947-1.890 | 0.099 |
| WHR | 8.059 | 0.668-97.180 | 0.100 |
| Fastglucose (mg/dL) | 1.004 | 1.001-1.008 | 0.022 |
| Lipoprotein[a] (mg/dL) | 1.007 | 1.003-1.012 | 0.002 |
| RHR (beats/min) | 1.011 | 0.996-1.026 | 0.146 |
| AVC degree | 9.417 | 6.694-13.247 | <0.001 |
