# Supplementary figures and images for: Construction and Verification of a Predictive Model for the Progression of Aortic Valve Calcification
Source: Glob Heart. 2025 Sep 24;20(1):84. doi: 10.5334/gh.1473 (PMC12466327; doi:10.5334/gh.1473)

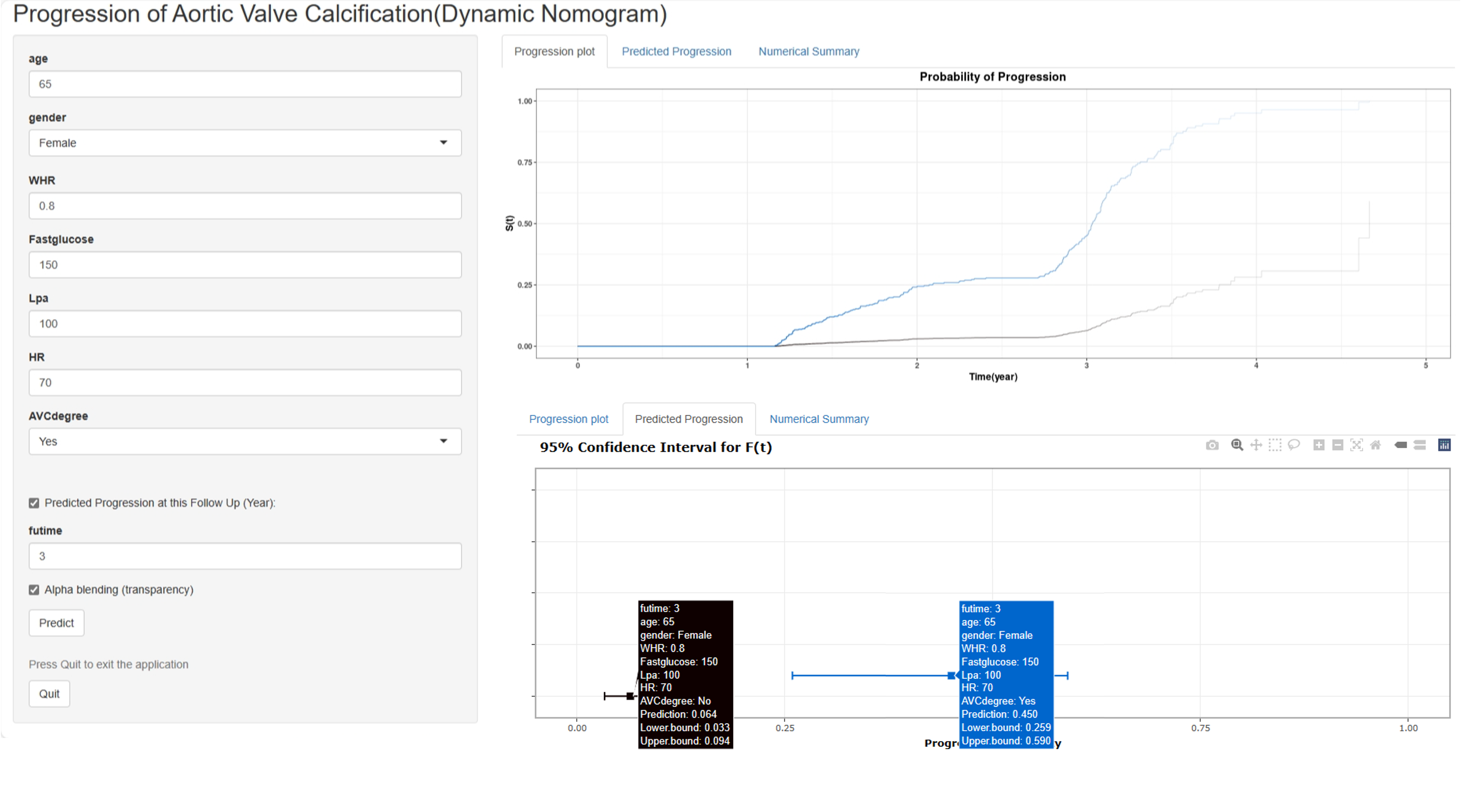

Supplement: Supplementary file. — Figure S1 and Tables S1 to S2. [file gh-20-1-1473-s1.zip › gh-1473_zhuang-s1/Figure S1.tif]
